# RNA-seq based gene expression analysis of ovarian granulosa cells exposed to zearalenone *in vitro*: significance to steroidogenesis

**DOI:** 10.18632/oncotarget.19699

**Published:** 2017-07-31

**Authors:** Guo-Liang Zhang, Rui-Qian Zhang, Xiao-Feng Sun, Shun-Feng Cheng, Yu-Feng Wang, Chuan-Liang Ji, Yan-Zhong Feng, Jie Yu, Wei Ge, Yong Zhao, Shi-Duo Sun, Wei Shen, Lan Li

**Affiliations:** ^1^ College of Animal Science and Technology, Qingdao Agricultural University, Qingdao Shandong 266109, China; ^2^ National Engineering Research Center for Gelatin-based Traditional Chinese Medicine, Dong-E-E-Jiao Co., Ltd, Liaocheng Shandong 252000, China; ^3^ College of Animal Science and Technology, Northwest A&F University, Yangling Shaanxi 712100, China; ^4^ Institute of Animal Sciences, Heilongjiang Academy of Agricultural Sciences, Harbin Heilongjiang 150086, China

**Keywords:** porcine, granulosa cells, steroidogenesis, gene expression, RNA-seq

## Abstract

Zearalenone (ZEA) is a natural contaminant of various food and feed products representing a significant problem worldwide. Since the occurrence of ZEA in grains and feeds is frequent, the present study was carried out to evaluate the possible effects of ZEA on steroid production and gene expression of porcine granulosa cells, using RNA-seq analysis. Porcine granulosa cells were administered 10 μM and 30 μM ZEA during 72 h of culture *in vitro*. Following ZEA treatment the gene expression profile of control and exposed granulosa cells was compared using RNA-seq analysis. The results showed that in the exposed granulosa cells ZEA significantly altered the transcript levels, particularly steroidogenesis associated genes. Compared with the control group, 10 μM and 30 μM ZEA treatment significantly increased the mRNA expression of *EDN1*, *IER3*, *TGFβ* and *BDNF* genes and significantly reduced the mRNA expression of *IGF-1* and *SFRP2* genes. In particular, ZEA significantly decreased the expression of genes essential for estrogen synthesis including *FSHR*, *CYP19A1* and *HSD17β* in granulosa cells. Furthermore, Q-PCR and Western-blot analysis also confirmed reduced expression of these genes in ZEA exposed granulosa cells. These effects were associated with a significant reduction of 17β-estradiol concentrations in the culture medium of granulosa cells. Collectively, these results demonstrated a concretely deleterious effect of ZEA exposure on the mRNA expression of steroidogenesis related genes and the production of steroid hormones in porcine ovarian granulosa cells *in vitro*.

## INTRODUCTION

Zearalenone (ZEA) is a toxic compound produced by several species of *Fusarium* and causing mycotoxicosis in animals [1]. In the past few years, reports on the metabolism of ZEA in mammals were published. Because of its estrogenic activity, ZEA could cause reproductive disorders in domestic animals and estrogenic syndromes in humans [2].

Pigs appear to be more sensitive to the exposure of dietary ZEA than other animals [1]. In young swine, after a single oral administration, ZEA is rapidly absorbed and metabolized *in vivo*. The absorption of ZEA in swine was estimated to be 80 - 85 % following administration of an oral dose of 10 mg/kg bodyweight [3]. ZEA has been detected in the follicular fluid of porcine antral follicles using liquid chromatography tandem mass spectrometry, and the concentrations of α-ZEA and ZEA in porcine follicular fluid were 17.6 pg/ml and 38.9 pg/ml respectively [4]. The effects of ZEA on weaned and prepubertal gilts have been described as vulvar and uterine hypertrophy, ovarian atrophy and mammary enlargement [5]. ZEA causes sterility in sows through inciting disorders of the ovary [6]. The oocytes die in primordial follicles, and despite having signs of estrus there is no ovulation [7]. Moreover, ZEA and its derivatives act similarly to 17β-estradiol (E_2_) in inhibiting the secretion and release of steroid hormones, thus disrupting endogenous estrogenic response during the preovulatory stage and depressing the maturation of ovarian follicles [8]. Changes in the estrous cycle, induced by ZEA, depend on the dose and time of administration [8].

The mammalian ovaries are female reproductive organs consisting of oocytes, granulosa and theca cells. Follicle growth is based on the growth of granulosa cells and oocytes in the early stages, and steroidogenesis is also accelerated by follicle development [9, 10]. Granulosa cells surround oocytes and produce ovarian steroid hormones, including estrogen and progesterone [11]. Ovarian steroids are essential for the function and normal development of several organs, including the uterus, mammary glands and the brain. In addition, ovarian steroids have local effects that are critical for maintaining normal ovarian physiology. Steroidogenesis, the pathway of steroid hormone biosynthesis, begins with cholesterol as the initial substrate. In the majority of mammals, steroidogenesis appears to occur via the two cell/two gonadotropin model in which androgens are synthesized from cholesterol by luteinizing hormone (*LH*) responsive theca cells, followed by conversion to estrogens in follicle stimulating hormone (*FSH*) exposed granulosa cells [12]. The *FSH* receptor (*FSHR*) is widely known as a representative biomarker during the folliculogenesis [13, 14]. The first stage of steroidogenesis is steroidogenic acute regulatory protein (*STAR*)-mediated transportation of cholesterol into the mitochondria. *LH* receptor (*LHR*) and the enzyme 17α-hydroxylase (*CYP17A1*), which converts pregnenolone by 3β-hydroxysteroid dehydrogenase (*HSD3β*) and progesterone to dehydroepiandrosterone and androstenedione, respectively, are expressed primarily in theca cells, while *FSHR* and 19α-hydroxylase (*CYP19A1*), which convert androgens to estrogens, are expressed mainly in granulosa cells [15].

Porcine follicular atresia caused by granulosa cell apoptosis may be one of the most dominant factors affecting prepubertal porcine reproduction. Previous studies indicated that a high concentration (≥ 60 μM) of ZEA could induce apoptosis in porcine granulosa cells as a result of disrupting porcine ovarian steroidogenesis [16]. Interestingly, our previous results demonstrated that low concentration (10 μM) ZEA exposure may affect the functions of the ovary, such as female germ cell cyst breakdown, and primordial follicle formation and development [17]. The present study utilized RNA-seq analysis to verify whether ZEA exposure can affect the mRNA expression and steroidogenesis of granulosa cells *in vitro*.

## RESULTS

### ZEA exposure affects granulosa cell development and gene expression

**Figure 1:**
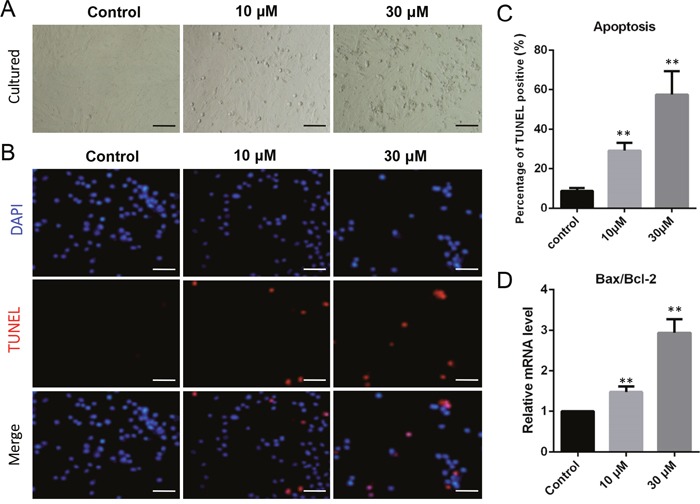
ZEA exposure increased cell apoptosis and induced the apoptosis-related gene mRNA abundance in cultured granulosa cells **(A)** Cultured granulosa cells *in vitro*. **(B)** Immunofluorescent staining of TUNEL and DAPI. Bars indicate 50 μm. **(C)** Graph indicating the percentages of TUNEL positive granulosa cells. **(D)** The mRNA levels of *Bax/Bcl-2* in cultured granulosa cells exposed to ZEA and control groups as determined by Q-PCR. The results are presented as mean ± SD. All experiments were repeated at least three times. * P < 0.05; ** P < 0.01.

Porcine granulosa cells were cultured *in vitro* and exposed to 10 μM or 30 μM ZEA for 72 h (Figure 1A). The percentages of TUNEL positive granulosa cells significantly increased following ZEA exposure (10 μM: 29.07 ± 0.78 %; 30 μM: 57.47 ± 0.93 %) compared with that of the control group (0 μM: 8.81 ± 0.57 %; *P* < 0.01; Figure 1B–1C). As shown in Figure 1D, the ratios of *Bax/Bcl-2* mRNA expression significantly increased in 10 μM (1.49 ± 0.19 fold) and 30 μM (2.94 ± 0.41 fold) ZEA-exposed granulosa cells compared with that of the control group (*P* < 0.01).

**Figure 2:**
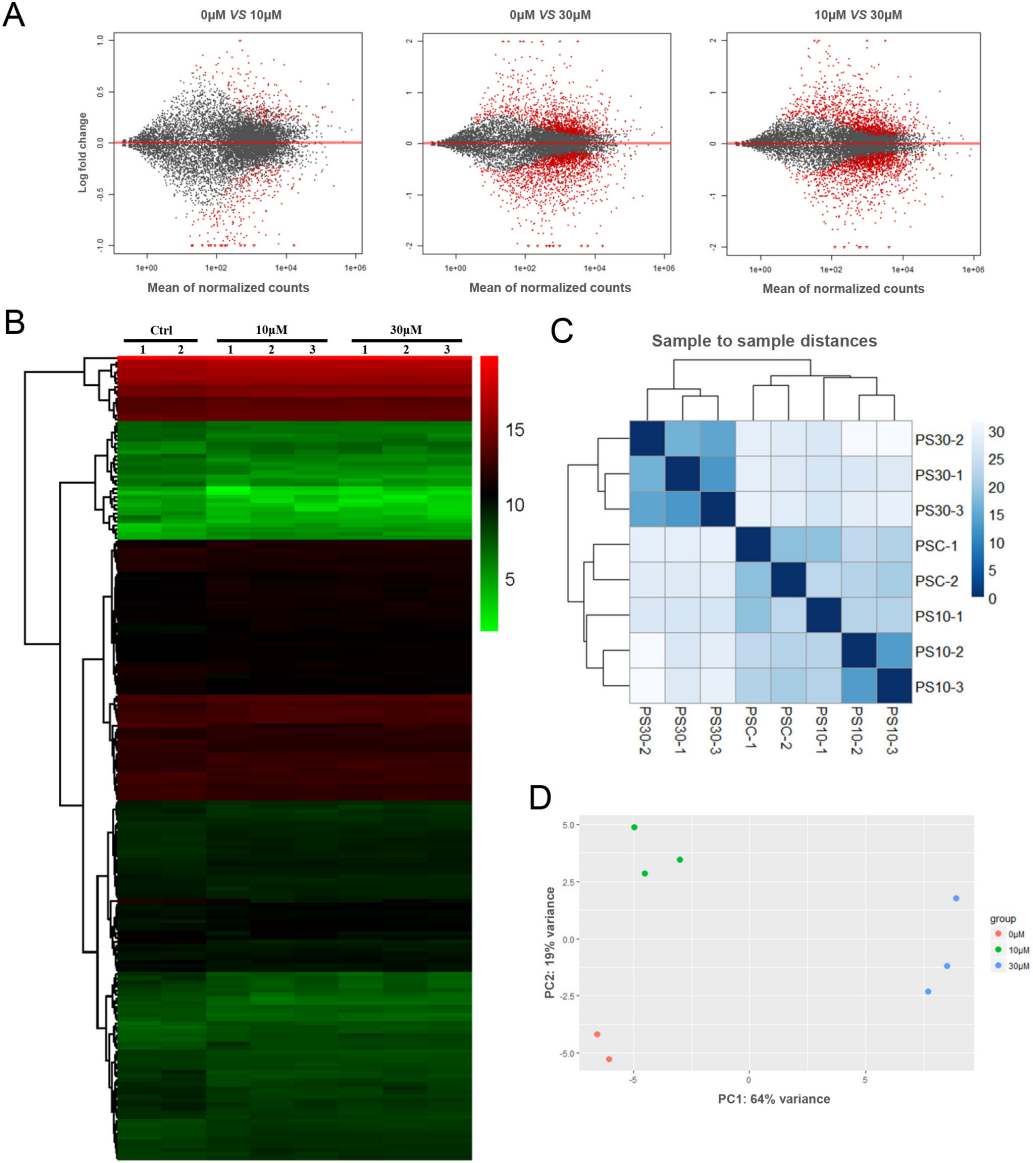
Granulosa cell gene expression profiling after ZEA treatment **(A)** Scatterplot of gene expression after ZEA treatment. Control group *vs*. 10 μM ZEA-treatment group, Control group *vs*. 30 μM ZEA-treatment group, 10 μM ZEA-treatment group *vs*. 30 μM ZEA-treatment group. Red points represent genes expressed significantly different. **(B)** Heatmap indicating the group differences of DEGs in the 10 μM and 30 μM ZEA-treated groups compared with the control group, and the repeatability within each group. **(C)** Sample-to-sample distances. Heatmap showing the Euclidean distances between the samples as calculated from the normalized log transformation. **(D)** PCA plot. The 8 samples shown in the 2D plane spanned by the first three principal components. The results are presented as mean ± SD. All experiments were repeated at least three times.

To analyze the effects of ZEA exposure on porcine granulosa cells, RNA-seq of granulosa cells was performed. According to the research criterion FDR (False Discovery Rate) < 0.1, a total of 25 324 genes were screened, including 6 374 differentially expressed genes (DEGs) between control and ZEA treatment groups, 2 926 DEGs between 10 μM and 30 μM ZEA treatment groups (Figure 2A). The number of DEGs caused by 10 μM and 30 μM ZEA were 381 and 1979 respectively. We obtained three repeats of expressed genes from 0 μM, 10 μM and 30 μM ZEA-treated granulosa cells RNA-seq data. One repeat of control groups was abandoned with a wide variation (Figure 2C–2D). Then the heat map was produced from the results of DEGs (Figure 2B). In this study, we chose the DEGs whose degree were greater than 20 from 0 μM group *vs* 10 μM group, and 0 μM group *vs* 30 μM group, respectively.

**Figure 3:**
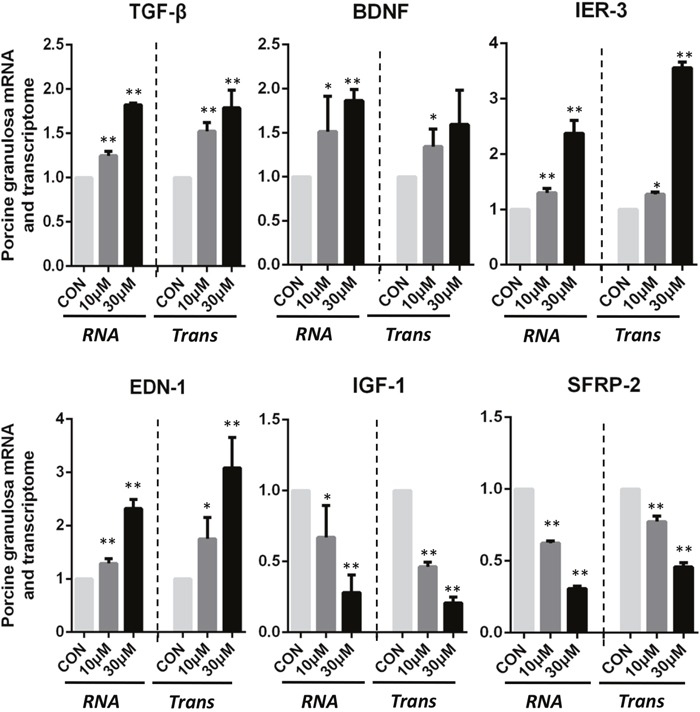
ZEA exposure affected the mRNA abundance of genes in the cultured granulosa cells Granulosa cells incubated with ZEA for 72 h. Quantitative RT-PCR for *TGF-β, BDNF, IER-3, EDN-1, IGF-1* and *SFRP-2* transcription factors. The mRNA level of all genes was normalized to the porcine granulosa cells *GAPDH* gene. The results are presented as mean ± SD. All experiments were repeated at least three times. * P < 0.05; ** P < 0.01.

There were 6 genes selected from the DEGs between control and ZEA-treated groups to verify the RNA-seq analysis data. As shown in Figure 3, ZEA exposure significantly down-regulated the mRNA abundance of Insulin Like Growth Factors 1 (*IGF-1*) and Secreted Frizzled Related Protein 2 (*SFRP-2*) genes, and significantly up-regulated the mRNA levels of Transforming Growth Factor Beta Receptor (*TGF-β*), Brain Derived Neurotrophic Factor (*BDNF*), Immediate Early Response 3 (*IER-3*), and Endothelin 1 (*EDN-1)* genes compared to that of the control groups (*p-value* < 0.05, *q-value* < 0.1), and the results of the Q-PCR were similar to those of the RNA-seq data analysis.

To explore the possible mechanism of ZEA exposure on porcine granulosa cells, we used the Search Tool for the Retrieval of Interacting Genes (STRING) database to annotate functional interactions between DEGs of the control and ZEA-treated groups. Based on this information, a protein-protein interaction (PPI) network was visualized by Cytoscape (Supplementary Figure 2).

Due to the complex and large number of the protein interactions, the DEGs with greater than 20 degree were selected between the control and ZEA-treated groups to visualize the sub-network of the PPI network (Supplementary Figure 2). Analyzing the center nodes of the networks we observed that in the 0 μM and 10 μM ZEA-treated groups, the Cyclin B (*CCNB*) encoded protein interacted with most of the DEG encoded proteins predicting strong interaction with 87 of these proteins. The remaining key nodes of the network were the Polo like Kinase-1 (*PLK-1*), *EDN-1*, *IGF-1*, SMAD Family Member (*SMAD-6*) and *FSHR* (Supplementary Figure 2A). The number of interactions (degree) were 70, 52, 41, 50 and 48, respectively. In the network of 0 μM and 10 μM ZEA-treated groups, *TGF-β* was the protein with the highest interactions, showing strong interactions with 102 DEG encoded proteins. The remaining key nodes were *Bcl-2* Associated X Protein (*BAX*), *EDN-1*, *IGF-1*, and the degree were 63, 87 and 95, respectively (Supplementary Figure 2B).

### DEGs involved in gene ontology (GO) classification and kyoto encyclopedia of genes and genomes (KEGG) pathways

**Figure 4:**
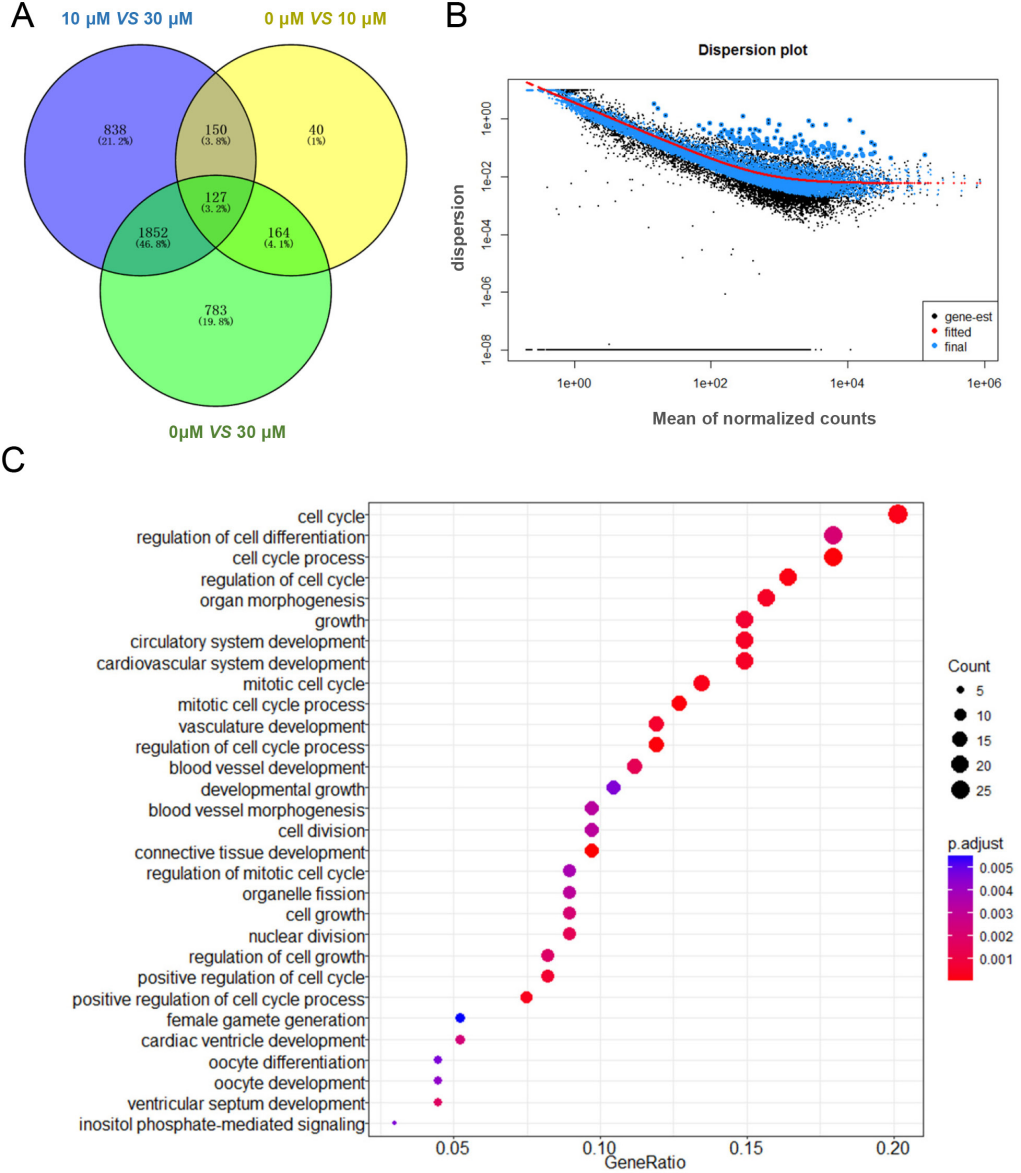
Gene expression of RNA-seq analysis in granulosa cells between control and ZEA-treated groups **(A)** Venn diagram showed that the different expression of 3 954 genes in control and ZEA-treated groups. **(B)** Dispersion plot. The dispersion estimate plot shows the gene-wise estimates (black), the fitted values (red), and the final maximum a posteriori estimates used in testing (blue). **(C)** Scattergram of overrepresented GO terms in biological process categories.

We obtained three groups of DEGs by comparing each group from the 0 μM, 10 μM and 30 μM ZEA-treated granulosa cells. Then Venn diagrams were constructed by the results. The number of shared DEGs caused by 10 μM and 30 μM ZEA treatment was 164 (Figure 4A). We used DAVID to identify GO-enriched functions for the 164 DEGs (Figure 4B). The DEGs from the granulosa cells treated with ZEA were significantly enriched in cell cycle, cell cycle process, regulation of cell cycle, mitotic cell cycle, cell growth and mitotic cell cycle process (Figure 4C). These functions were significantly associated with cell cycle and metabolic regulation of granulosa cells. In addition, the downregulation of DEGs from the granulosa cells treated with ZEA were significantly enriched in cell cycle (p < 1.66E-5), mitosis (p < 7.97E-5), and metabolic process (p < 0.000182) (Table 1).

**Table 1:** Analysis of Gene Ontology (GO) biological process of differentially expressed genes (DEGs)

GO ID	Description	Term type	p_value	q_value	count
GO:0030282	bone mineralization	biological_process	9.02E-06	0.007386	5
GO:0007049	cell cycle	biological_process	1.66E-05	0.007386	7
GO:0031214	biomineral tissue development	biological_process	2.62E-05	0.007751	5
GO:0007067	Mitosis	biological_process	7.97E-05	0.016073	5
GO:0009887	organ morphogenesis	biological_process	9.25E-05	0.016073	10
GO:0000904	cell morphogenesis involved in differentiation	biological_process	0.000109	0.016073	8
GO:0044236	multicellular organism metabolic process	biological_process	0.000182	0.023138	3

The R package of clusterProfiler was used to determine profoundly affected KEGG pathways in order to obtain further insights into the function of the DEGs. A total of the control and ZEA treatment group DEGs, containing 114 genes down-regulated and 50 genes up-regulated in the ZEA exposure granulosa cells, were selected. On the basis of the results, the downregulated DEGs were significantly enriched in the *TGF-β* signaling pathway (p = 0.000737, q-value = 0.030), Gap junction (p = 0.001254, q-value = 0.030), and *GnRH* signaling pathway (*p-value* = 0.001306, *q-value* = 0.030) (Table 2). In addition, we obtained 20 core DEGs from the enriched pathway (Supplementary Figure 3, Table 3). Finally, by performing the KEGG pathway analysis of DEGs we identified 3 genes involved in steroidogenesis of granulosa cells that are downregulated including *FSHR*, *CYP19A1* and *HSD17β* (Supplementary Figure 3, Table 3).

**Table 2:** Analysis of Kyoto Encyclopedia of Genes and Genomes (KEGG) pathways of DEGs

KEGG ID	Description	GeneRatio	p_value	q_value	count
ssc04970	Salivary secretion	5/40	4.62E-05	0.004278	5
ssc04350	TGF-β signaling pathway	4/40	0.000737	0.030238	4
ssc04540	Gap junction	4/40	0.001254	0.030238	4
ssc04912	GnRH signaling pathway	4/40	0.001306	0.030238	4

**Table 3:** Core genes expression of RNA-seq analysis in granulosa cells between control and 10 μΜ ZEA-treated groups

Gene id	Sample 0μM	Sample 10μM	Log_2_FoldChange	p_value	q_value
*TGF-β*	32.7635	53.1452	−0.69785	5.00E-05	0.000932
*BDNF*	27.2512	36.5626	−0.42405	0.0019	0.021078
*SFRP-2*	325.921	237.694	0.455414	5.00E-05	0.000932
*IGF-1*	30.507	13.9171	1.13228	5.00E-05	0.000932
*EDN-1*	15.2035	26.63	−0.80865	5.00E-05	0.000932
*IER-3*	13.9081	17.983	−0.37071	0.00545	0.048266

### ZEA exposure affects porcine granulosa cell steroidogenesis

We screened the frequent DEG encoded protein interactions of the PPI network using the STRING (http://string-db.org/) analysis tool. In Supplementary Figure 4, several PPI nodes had higher degrees, as follows: *TGF-β* (degree = 43), *IGF-1* (degree = 26), *EDN-1* (degree = 24), *IER-3* (degree = 24). The results indicated that these proteins directly or indirectly interacted with other DEGs and regulated the DEGs functions. The most frequent DEG encoded protein interactions shared by the networks are shown in Supplementary Figure 5. There were 3 hub genes with higher degrees, including *FSHR* (degree = 31), *CYP19A1* (degree = 27), and *HSD17β* (degree = 27). Notably, *HSD17β*, *CYP19A1* and *FSHR* were involved in direct or indirect interaction with steroidogenesis (Supplementary Figure 5). These enzymes or enzyme-related proteins on steroidogenesis were expressed in ovarian granulosa cells. The E_2_ would be inhibited with the reduction of these steroidogenic enzymes.

**Figure 5:**
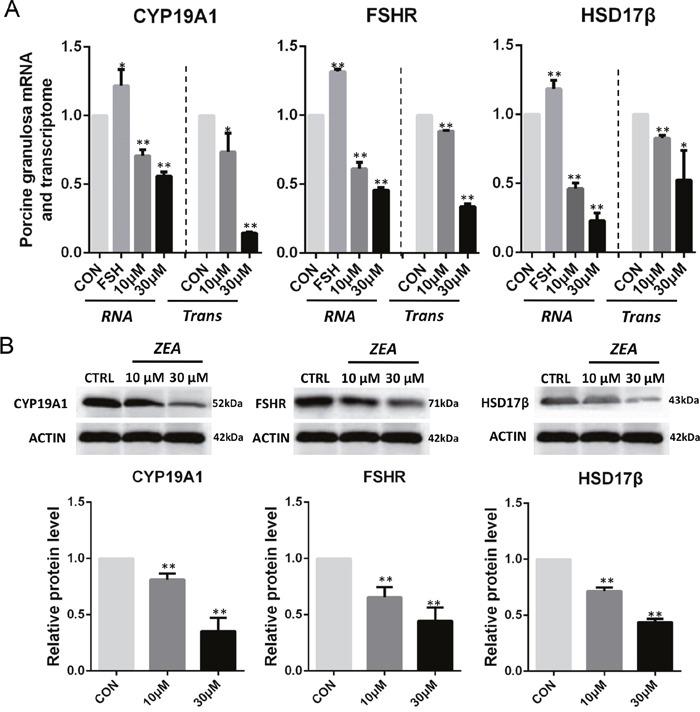
ZEA exposure affected the mRNA and protein abundance of steroidogenesis-related genes in cultured granulosa cells **(A)** Quantitative RT-PCR for *CYP19A1*, *FSHR*, and *HSD17β* transcription factors. The mRNA levels of all genes was normalized to the porcine granulosa cells *GAPDH* gene. **(B**) Protein levels of *CYP19A1/ACTIN*, *FSHR/ACTIN*, and *HSD17β/ACTIN* by western blotting. The protein levels was normalized to *ACTIN*. The results are presented as mean ± SD. All experiments were repeated at least three times. *P < 0.05; ** P < 0.01.

On the basis of the results reported above we predicted that ZEA exposure impacted the steroidogenesis of granulosa cells by causing downregulation of transcripts for critical enzymes involved in the estrogen pathway. To verify such a possibility, we compared the expression level of several transcript factors and enzymes involved in the pathway in the control and ZEA-treated granulosa cells both at the mRNA and protein level. Q-PCR analyses showed that ZEA exposure significantly down-regulated the mRNA abundance of *FSHR*, *CYP19A1* and *HSD17β* genes (*P* < 0.05 or *P* < 0.01; Figure 5A). As shown in Figure 5B, ZEA-treated granulosa cells had lower protein levels of *FSHR* (10 μM: 0.65 ± 0.02; 30 μM: 0.44 ± 0.03), *CYP19A1* (10 μM: 0.81 ± 0.03; 30 μM: 0.35 ± 0.02) and *HSD17β* (10 μM: 0.71± 0.04; 30 μM: 0.43 ± 0.04) than that of the control granulosa cells (*P* < 0.05 or *P* < 0.01).

**Figure 6:**
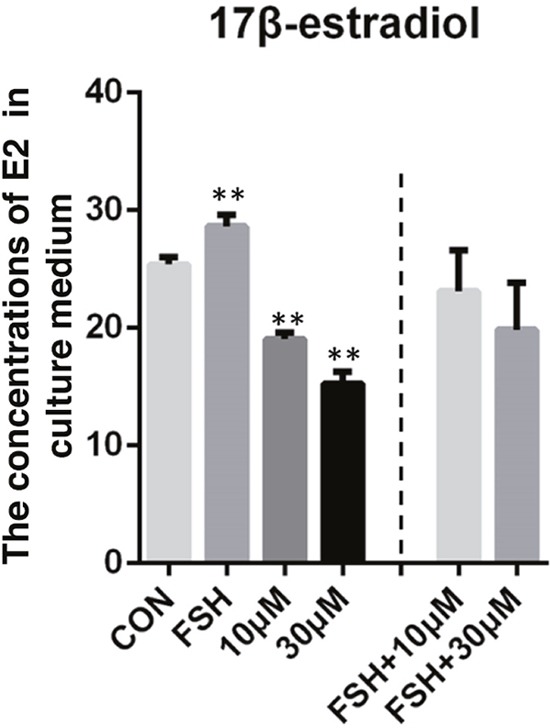
17β-estradiol (E_2_) level in granulosa cells-culture medium ZEA exposure resulted in a significant reduction in the concentrations of E_2_ in granulosa cells-culture medium and could be rescued by FSH. The results are presented as mean ± SD. All experiments were repeated at least three times.* P < 0.05; ** P < 0.01.

Interestingly, compared to the control groups, the co-treatment of 5 μg/ml FSH and ZEA (10 μM or 30 μM) treatment groups showed no significantly decreased level of E_2_ in the granulosa cells, while the 10 μM and 30 μM ZEA treated groups showed significantly decreased level of E_2_. In addition, 5 μg/ml FSH treatment significantly increased the level of E_2_ in the granulosa cells. These results indicated that FSH might rescue the decreased level of E_2_ observed in the ZEA treatment groups (*P* < 0.05 or *P* < 0.01; Figure 6).

**Figure 7:**
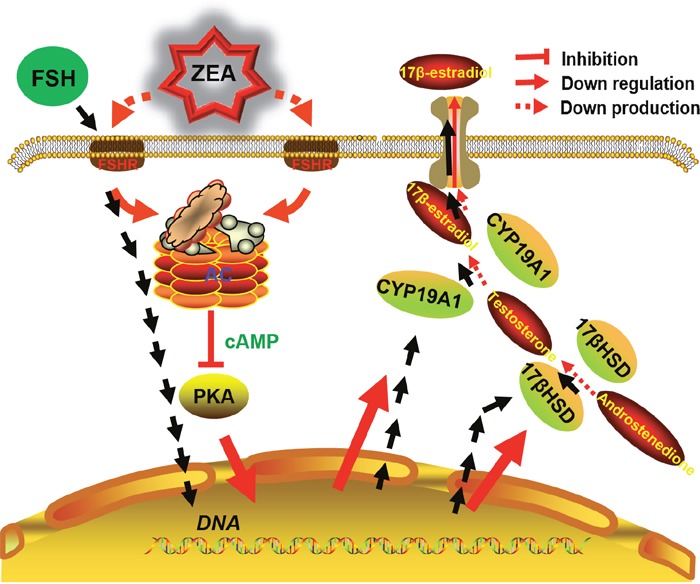
The diagram showed steroidogenesis pathways affected by FSH and the likely mechanisms for decreasing steroidogenesis induced by ZEA-exposure in granulosa cells

In addition, we propose a possible mechanism of ZEA exposure disrupting the steroidogenesis by DEGs enriched in the pathway of ovarian steroidogenesis and the results of ZEA exposure on the granulosa cells (Figure 7).

## DISCUSSION

Numerous studies have focused on the effects of endocrine disrupting chemicals (EDCs) on female reproduction. We previously found that ZEA led to apoptosis of ovarian germ cells, altered gene expression and impaired the formation of primordial follicle in the newborn mouse [17]. We suspected that ZEA altered the development and steroidogenesis of ovarian granulosa cells, which in turn caused indirect effects on mammalian fertility. To our knowledge, there is little information about the comprehensive molecular mechanisms of porcine granulosa cells development and steroidogenesis influenced by ZEA.

Using RNA-seq analysis, we found that ZEA exposure significantly altered the mRNA expression of hundreds of genes in the granulosa cells. In particular, *TGF-β* and *SMAD-7* genes were found to be upregulated and downregulated, respectively, suggesting ZEA exposure is involved in the antiproliferative response to *TGF-β* [18]. For example, *TGF-β* induces the dephosphorylation of *p70S6K* by *PP2A*, which leads to cell cycle arrest [19]. With the regulation of the cell cycle or cell differentiation, *TGF-β* and *SMAD-7* can trigger apoptosis. Mechanisms of *TGF-β* induced apoptosis include an increase in the expression of death associated protein kinase *DAPK* [20] and the binding of the proapoptotic effector to *Bcl-2* in gastric carcinoma cell lines [21]. In addition, Zhu et al. have demonstrated that high concentrations of ZEA (> 60 μM) increases reactive oxygen species (ROS) levels, decreases the proliferation of porcine granulosa cells and causes an obvious apoptosis and necrosis in porcine granulosa cells [16]. Our results indicated that low concentrations (10 μM) of ZEA exposure may increase ROS production via the *TGF-β* pathway by repressing the expression of antioxidant genes or by activating the expression of NADPH oxidase (*Nox*) [22, 23]. *TGF-β* induced ROS production can promote granulosa cell apoptosis through a mitochondrial-dependent pathway, or at least partially through the modulation of the *Bcl-2* family [24]. These results, together with our previous studies, suggest that ZEA may induce the apoptosis of granulosa cells via disrupting the *TGF-β* pathway [17, 19, 23].

Bioinformatics analyses confirmed that *IGF-I* and steroidogenesis related genes were particularly affected in the ZEA exposed granulosa cells. A previous study showed that *FSHR* and *IGF-I* gene are expressed in the granulosa cells of healthy growing follicles in the ovary [25]. The reduction in *FSHR* mRNA and aromatase expression was detected in the follicles of *IGF-I* knockout mice. Moreover, the reduced level of granulosa cell *FSHR* expression in *IGF-I* knockout ovaries led to the infertility in the female [26]. Interestingly, we found ZEA exposure down regulated the expression of *IGF-I* and *FSHR* genes which was the regulator of enzymes involved in steroidogenesis. However, local *IGF-I* expression is not essential for induction of granulosa cell *FSHR* gene expression *de novo*, since *FSHR* mRNA is still present, albeit at low levels, in the *IGF-I* knockout ovary [26]. *IGF-I* was related to the production of certain proteins that stabilize the *FSHR* mRNA in granulosa cells [27]. It is possible that ZEA has a primary effect on granulosa cell *FSHR* expression, and that *IGF-I* secondarily reduces *FSHR* expression to decrease the response to FSH.

On the other hand, most of the effects of endocrine disrupters on female reproduction have been attributed to their capability to deregulate steroid signaling at various levels [1, 28, 29]. The production of ovarian steroids relies on a series of receptors and steroidogenic enzymes expressed in theca or granulosa cells [30]. In the present study we found that ZEA exposure caused decreased mRNA levels of genes encoding crucial players involved in steroidogenic pathways mainly in granulosa cells such as the receptor of FSH and the enzymes *CYP19A1* and *HSD17β*. Western-blot results confirmed decreased expression of *CYP19A1* and *HSD17β* proteins in the ZEA exposed granulosa cells. In addition, in line with previous *in vivo* and *in vitro* studies, we found that ZEA decreased the level of ovarian PROG, E_2_ and ASD. A direct inhibition of the transcription of aromatase genes and/or the enzyme activity via *FSH* plus *IGF-I* induced *CYP19A1* and *CYP11A1* mRNA abundance by ZEA may be the mechanism of these effects [31]. The alterations of granulosa cell mitochondria observed by us also supports the impact of ZEA on steroidogenesis [24]. Though ZEA did not appear to affect follicle dynamics, it might be the cause of the reduction of the systemic level of E_2_ observed in the exposed animals. Interestingly, ZEA exposure resulted in a significant reduction in the concentrations of E_2_ in granulosa cell-culture medium. In mammals the steroid hormones produced by growing follicles serve as feedback signals to the hypothalamus and pituitary gland in order to establish a hormonal regulatory axis [32].

As reported above, results of the present study indicated that ZEA had direct dose-dependent effects on granulosa cell steroidogenesis. It is probable that porcine granulosa cells are the main targets of the ZEA as reported in the present study. What's more, we have developed an innovative, integrated method to hunt novel hubs associated with ZEA exposure on granulosa cells at a low cost. Up-regulated hub genes *EDN-1*, *IER-3*, *TGF-β* and *BDNF*, down-regulated hub genes *IGF-1*, *SFRP-2*, and estrogen synthesis related genes such as *FSHR*, *CYP19A1* and *HSD17β* might play important roles in the toxic effects of ZEA. These interactions and direct ovarian effects could be an important mechanism of the pollutant ZEA in feedstuffs impacting reproductive performance in swine.

## MATERIALS AND METHODS

### Reagents

ZEA (Z2125-10 MG) was purchased from Sigma (St. Louis, MO). Dimethyl sulfoxide (DMSO), M-199 medium, penicillin, streptomycin and fetal bovine serum (FBS) were obtained from Gibco (Carlsbad, CA). Stock solutions of ZEA were prepared by dissolving ZEA in DMSO.

### Animals

The ovaries of mature sows were collected from Qingdao Fu Wan Pig Production Cooperation (Qingdao, China). Porcine ovaries were obtained from a local slaughterhouse and maintained at 30 - 37°C for isolation of granulosa cells. The procedures of animal handling were reviewed and approved by the Ethical Committee of Qingdao Agricultural University (agreement No. 2015-18).

### Isolation and culture of porcine granulosa cells

Granulosa cells were aspirated aseptically from antral follicles (about 4 mm diameter) using a 20 ml syringe (18-gauge needles) [16]. After standing for 15 min, the granulosa cells were centrifuged at 300 g for 5 min according to the methods previously described [23]. The blood cells in the supernatant were aspirated after rinsing with phosphate-buffered saline (PBS). Then the granulosa cells were cultured in M-199 medium supplemented with 10 % FBS and 1 % penicillin-streptomycin in a humidified incubator with 5 % CO_2_ at 37°C [33].

### Drug treatments

Granulosa cells were seeded into 6 cm culture dishes; at a density of 1×10^6^ cells per dish. To study the generation of mRNA and protein expression in the ZEA-treated granulosa cells, ZEA was added to the medium at final concentrations of 10 μM and 30 μM and the cells were incubated for 72 h. To investigate the protective mechanism of FSH against ZEA-induced intracellular damage, cells were treated with or without 5 μg/ml FSH for 72 h co-treated with ZEA. Control groups set up with DMSO at the same concentrations treated with ZEA is to guarantee the accuracy of experimental design.

### TUNEL staining

TUNEL BrightRed Apoptosis Detection Kit (Vazyme, A11302, Nanjing, China) was used to evaluate the granulosa cell apoptosis following the manufacturer's instructions. Briefly, after 72 h ZEA treatment, the granulosa cells were collected by digestion with collagenase (0.2 % in PBS) for 6 min and fixed in 4 % paraformaldehyde for 2 h. After dripped onto the slides, the cells were heated at 42°C for 1 h. Then the slides of granulosa cells were washed twice with PBS. After incubation with 60 μl TUNEL reaction mixture (Label Solution and Enzyme Solution; 5:1) following the manufacturer's recommendations, for 60 min at 37°C without light, the granulosa cells were stained with DAPI and photographed with a fluorescence microscope. Five different areas were randomly selected from each section to calculate TUNEL positive cells under microscope. The calculations were conducted at least three independent experiments.

### RNA extraction, reverse transcription, and RNA-Seq

Porcine granulosa cells were harvested and extracted for total RNA using an RNAprep pure MicroKit (Aidlab, RN07, Beijing, China) according to the manufacturer's protocols. And the cDNAs were synthesized using a TURE script first strand cDNA Synthesis Kit (Aidlab, PC1802, Beijing, China) as described [34]. The reaction program was: 40 min at 42°C, 65°C for 15 min, and finally a cooling step at 4°C. For RNA-Seq, control and treatment granulosa cells were collected after *in vitro* ZEA exposure for 72 h. In each group, we applied 3 independent samples to extract total RNA using TRIZOL and sequenced at Novogene (Beijing, China).

### Data preprocessing and identification of DEGs

The sequencing data is subjected to quality control to obtain clean data. The clean reads were compared to the Sus_scrofa10.2 reference genome using HISAT2 software. Using featureCounts software to obtain gene expression levels. The CONTROL-3 sample correlation of RNA-seq was found to be different to other CONTROL sample group. Remove this samples in order not to affect subsequent analysis. Use R package DESeq2 for differential expression analysis. The classical Hypergeometric distribution test was applied in our analysis to check the GO enrichment analysis and KEGG analysis in 3 groups. The Benjamini & Hochberg method [35] was used to adjust the raw p value into the false discovery rate (FDR). Adjusted *q-value* < 0.1 were regarded as the cutoff criterion for DEGs.

### GO enrichment analysis

Based on Gene Ontology Database (http://www.geneontology.org/), the functions of DEGs between control and ZEA-treated samples were analyzed via GO, which is a commonly used approach for functional enrichment studies of large-scale genes [36]. Use the R package clusterProfiler for GO enrichment analysis *p-value* < 0.01 and adjusted *p-value* < 0.1 were regarded as the cutoff criterion for GO enrichment analysis.

### Pathway enrichment analysis

KEGG is a bioinformatics database including biochemistry pathways [37]. R package clusterProfiler provides analytic ways for extracting biological meaning from a large list of genes [38]. The R package clusterProfiler was used for KEGG pathway enrichment analysis DEGs. Screaming some pathways were analyzed for significant differences. *p-value* < 0.01 and adjusted *p-value* < 0.1 were regarded as the cutoff criterion for KEGG enrichment analysis.

### PPI network construction and modules mining

As proteins seldom perform their functions alone, it is important to comprehend the interaction of these proteins by studying larger functional groups of proteins [39]. The STRING database [40] provides both experimental and predicted interaction information. We used the STRING database to annotate functional interactions between DEGs. The edges and nodes of the PPI network were so complicated [38] that further analysis was needed to expose the enriched functional modules of the PPI network using STRING (http://string-db.org/) process [41]. Based on this information, a PPI network was visualized by Cytoscape [42].

### Quantitative real-time PCR

Supplementary Table 1 listed the primers used in this study. A LightCycler 480 real-time PCR instrument (Roche LC480) was used to carry out amplification using a Light CyclerVR SYBR Green I Master (Roche, 04887352001, Germany). Amplification was performed in a 20 μl reaction solution following the manufacturer's recommendations, containing 2 μl cDNA, 10 μl of SYBR green master mix, 0.8 μl of primers (20 μM), and 7.2 μl of nuclease-free water. The PCR reaction conditions: 10 min at 95°C, followed by 45 cycles of 95°C for 10 s, 60°C for 30 s and finally a cooling step at 4°C. The mRNA abundance of reference gene was normalized to the following formula: 2^− (target gene CT value –reference gene CT value)^. Each amplification was done in triplicate and the gene expression was normalized using the standard curve generated with *GAPDH* as the reference gene. We provided the standard curve and primer efficiency of all the primers in the Supplementary Figure 1.

### Western blotting

Protein lysates isolated from control and ZEA exposed granulosa cells were used for western blotting analysis according to standard methods [43, 44]. The proteins from each group were separated by SDS-PAGE and were transferred onto PVDF membranes. Following blocking with 5 % BSA in Tris-buffered saline, pH 7.4, containing 0.05 % Tween-20 (TBST), the membranes were incubated with anti-*ACTIN* antibody (Abcam, ab8226), rabbit anti-*FSHR* antibody (Sangon, D120641), rabbit anti-*HSD17β* antibody (Sangon, D261765), rabbit anti-*CYP19A1* antibody (Sangon, D260102), at the concentration of 1.0 μg/ml, overnight at 4°C. Then the membranes were incubated at 37°C for 2 h with secondary antibodies (Beyotime, A0208) at a dilution of 1:1500 in TBST after washing three times in TBST. The band intensity was quantified using Actin as internal control and measured with IPWIN software.

### Dosage of sex steroid hormones

The levels of E_2_ was assessed in granulosa cell culture medium using ELISA kits (LanpaiBIO, China) following the manufactures’ protocol. All samples were run in triplicates, and all coefficient of linear regression meet a criterion (R2 > 0.92).

### Statistical methods

Data are represented as mean ± SD. Differences between the control and treatment groups were statistically determined by one-way analysis of variance (ANOVA) followed by Tukey test for multiple comparisons using Graph-Pad Prism analysis software (Graph-Pad Software, San Diego, CA). Results were considered statistically significant at *P* < 0.05.

## SUPPLEMENTARY MATERIALS FIGURES AND TABLE


