# Supplementary material for: RNA-seq based gene expression analysis of ovarian granulosa cells exposed to zearalenone in vitro: significance to steroidogenesis
Source: Oncotarget. 2017 Jul 31;8(38):64001–14. doi: 10.18632/oncotarget.19699 (PMC5609980; doi:10.18632/oncotarget.19699)
Supplement: Supplementary file 1 [file oncotarget-08-64001-s001.pdf]

# RNA-seq based gene expression analysis of ovarian granulosa cells exposed to zearalenone *in vitro*: significance to steroidogenesis

## SUPPLEMENTARY MATERIALS

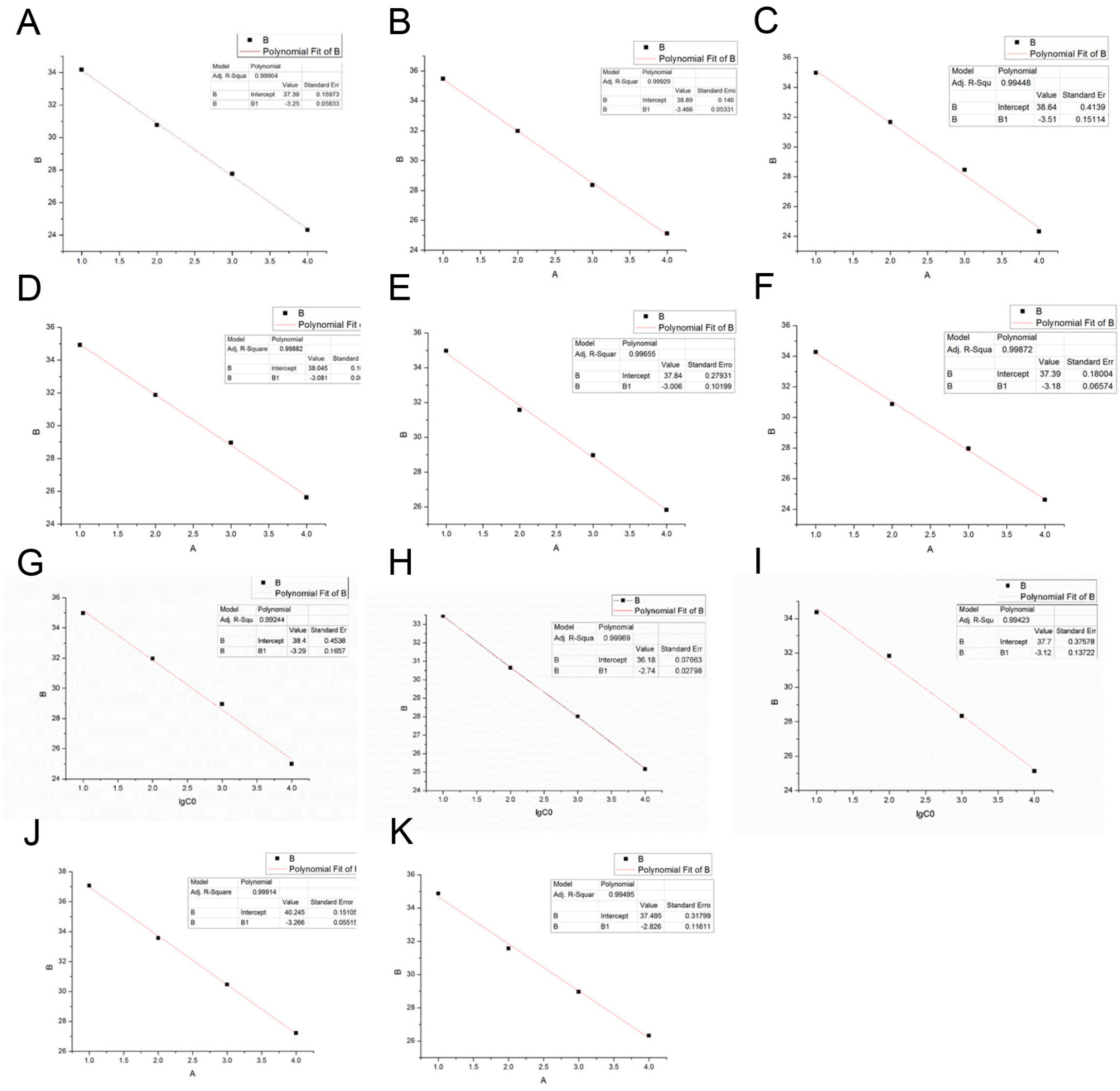

**Supplementary Figure 1: The standard curve and primer efficiency of all the Q-PCR primers.** (A-K) represent the standard curve and primer efficiency of *TGF-β, BDNF, IER-3, EDN-1, IGF-1, SFRP-2, CYP19A1, FSHR, HSD17β, Bax and Bcl-2* genes.

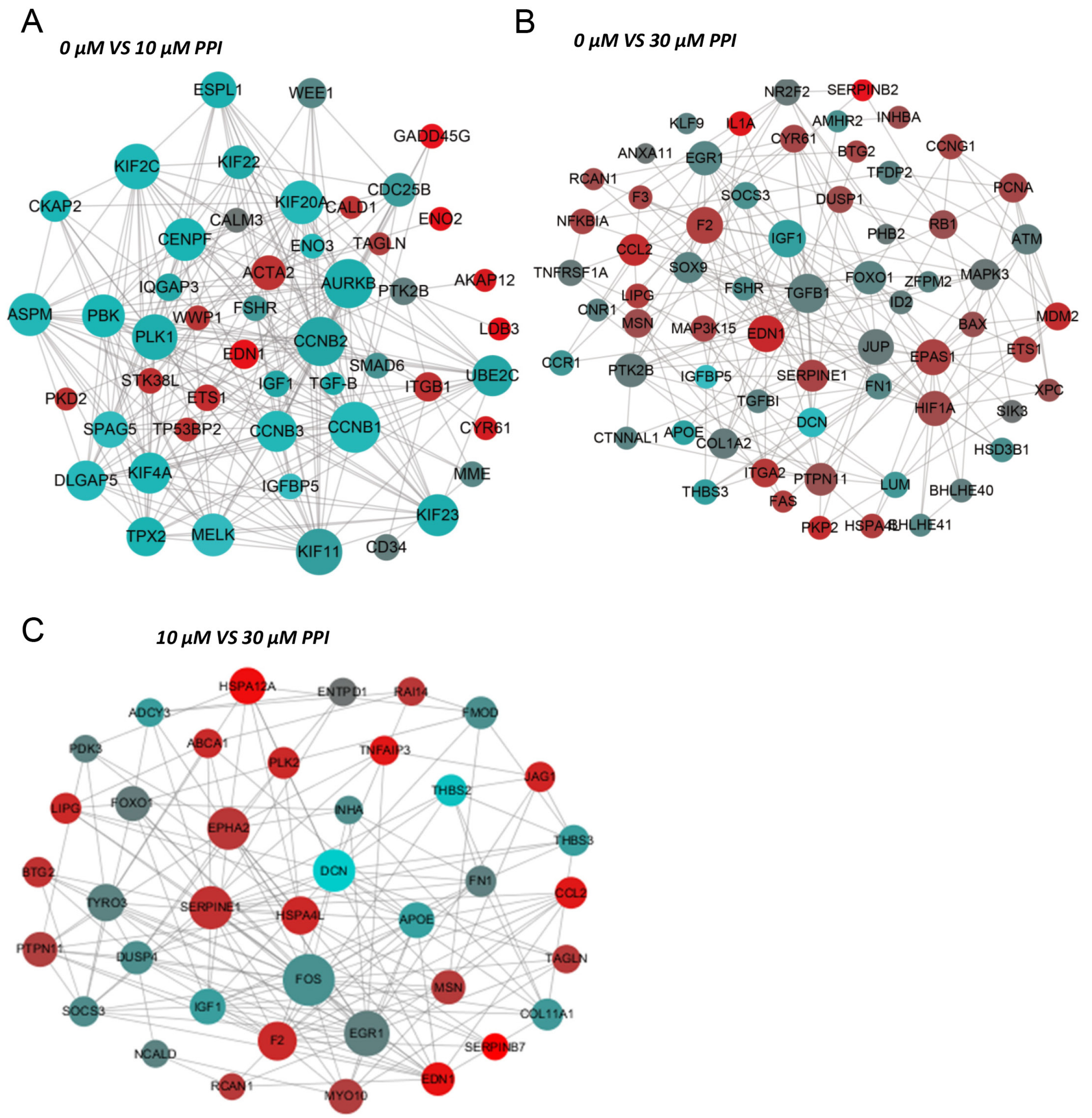


**Supplementary Figure 2: Protein-protein interaction (PPI) network based on the Search Tool for the Retrieval of Interacting Genes (STRING) database to annotate functional interactions between different expression genes in ZEA-treatment and control groups.** The 120 genes in 10 µM and 30 µM ZEA treatment group and node degree ≥20 was selected as the threshold. Red points represent genes up-regulated and blue points represent genes down-regulated in every two groups.

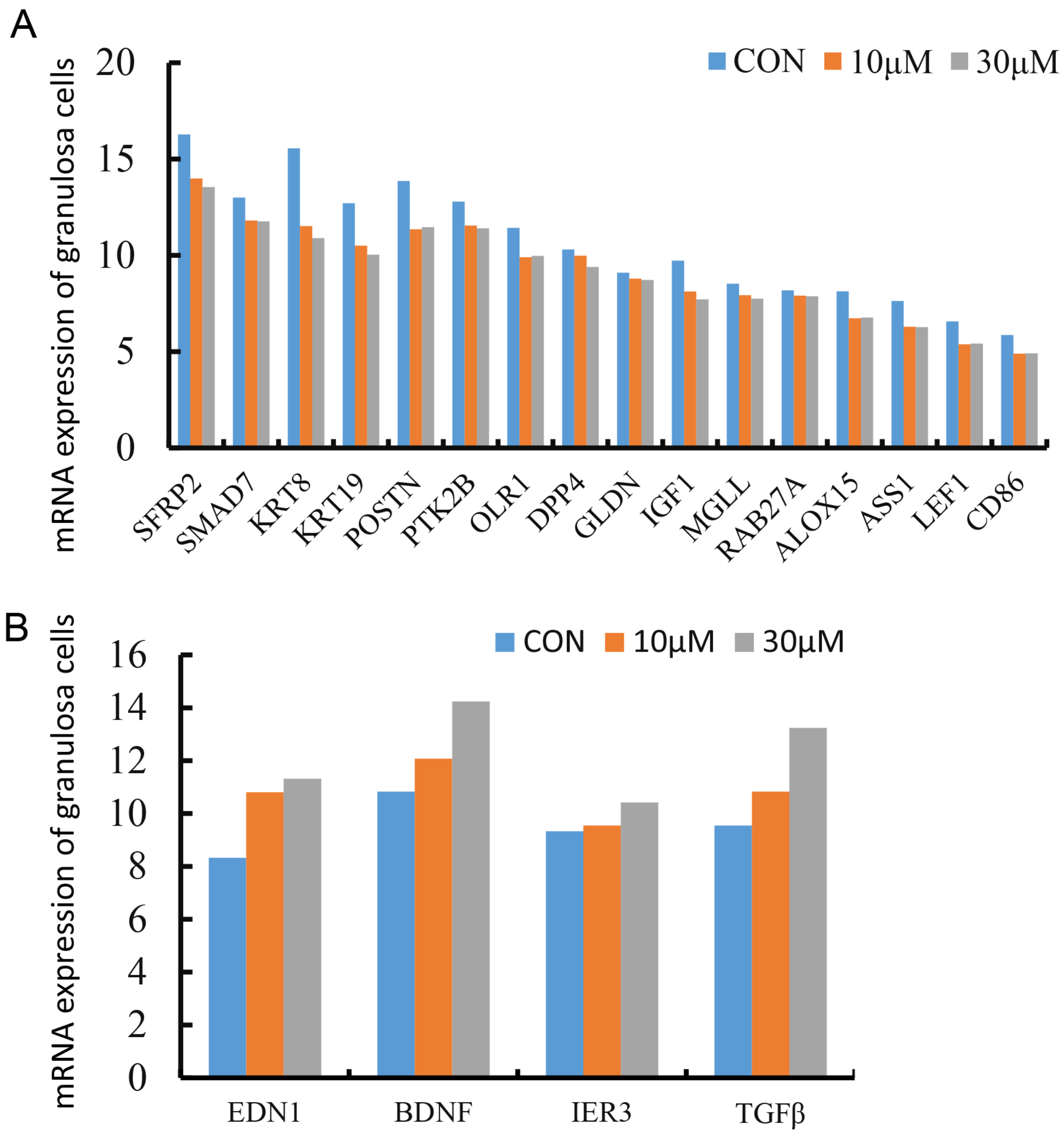


**Supplementary Figure 3: (A)** Part of declined expression genes between control and ZEA-treatment groups. **(B)** The increased expression genes in ZEA-treatment groups. Node degree ≥20 was selected as the threshold.

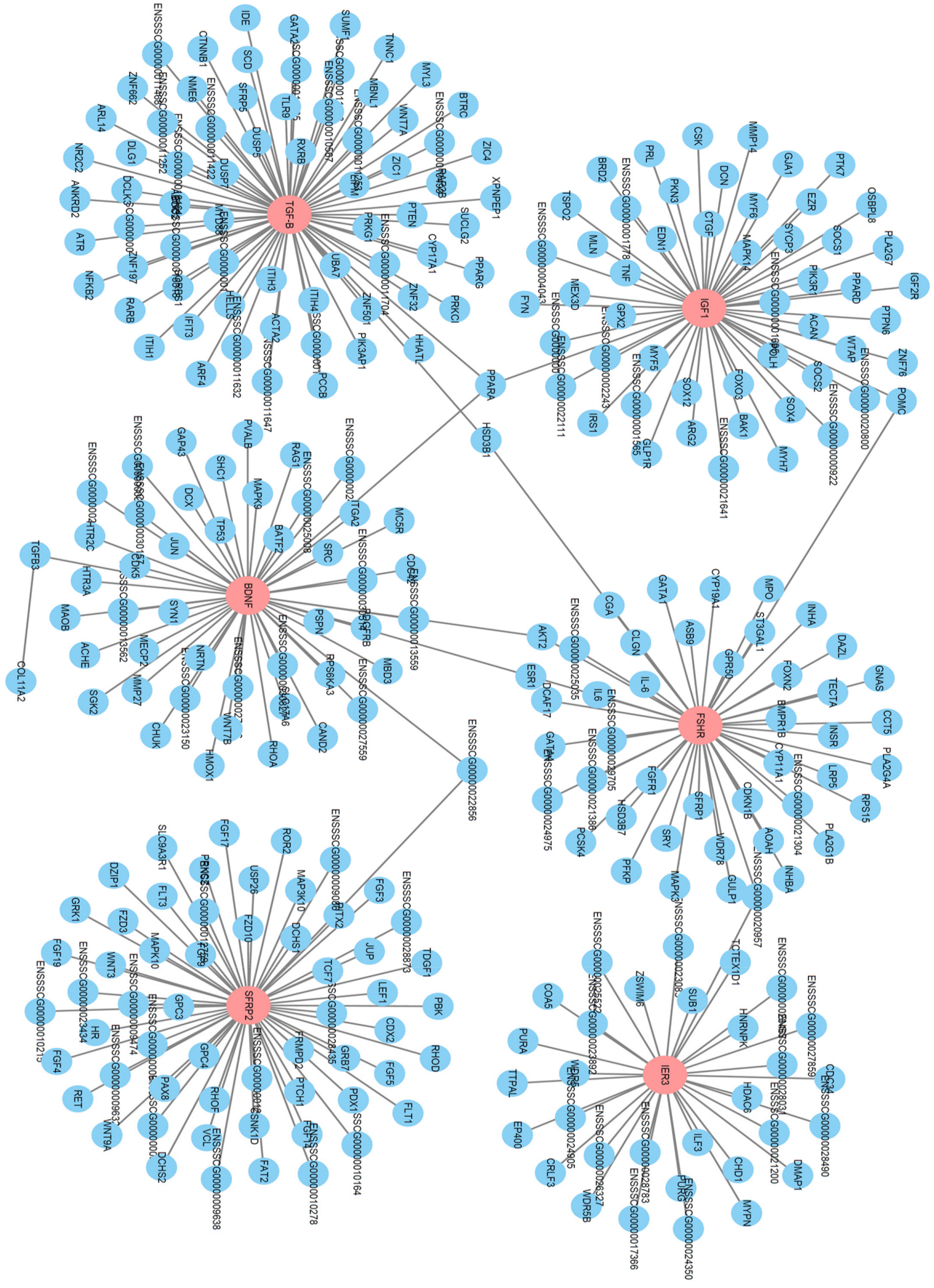


**Supplementary Figure 4: PPI network based on the STRING database to annotate functional interactions between apoptosis-related genes in ZEA-treatment groups.** We obtained 6 core differentially expression genes between control and ZEA-treated groups. Node degree ≥20 was selected as the threshold.

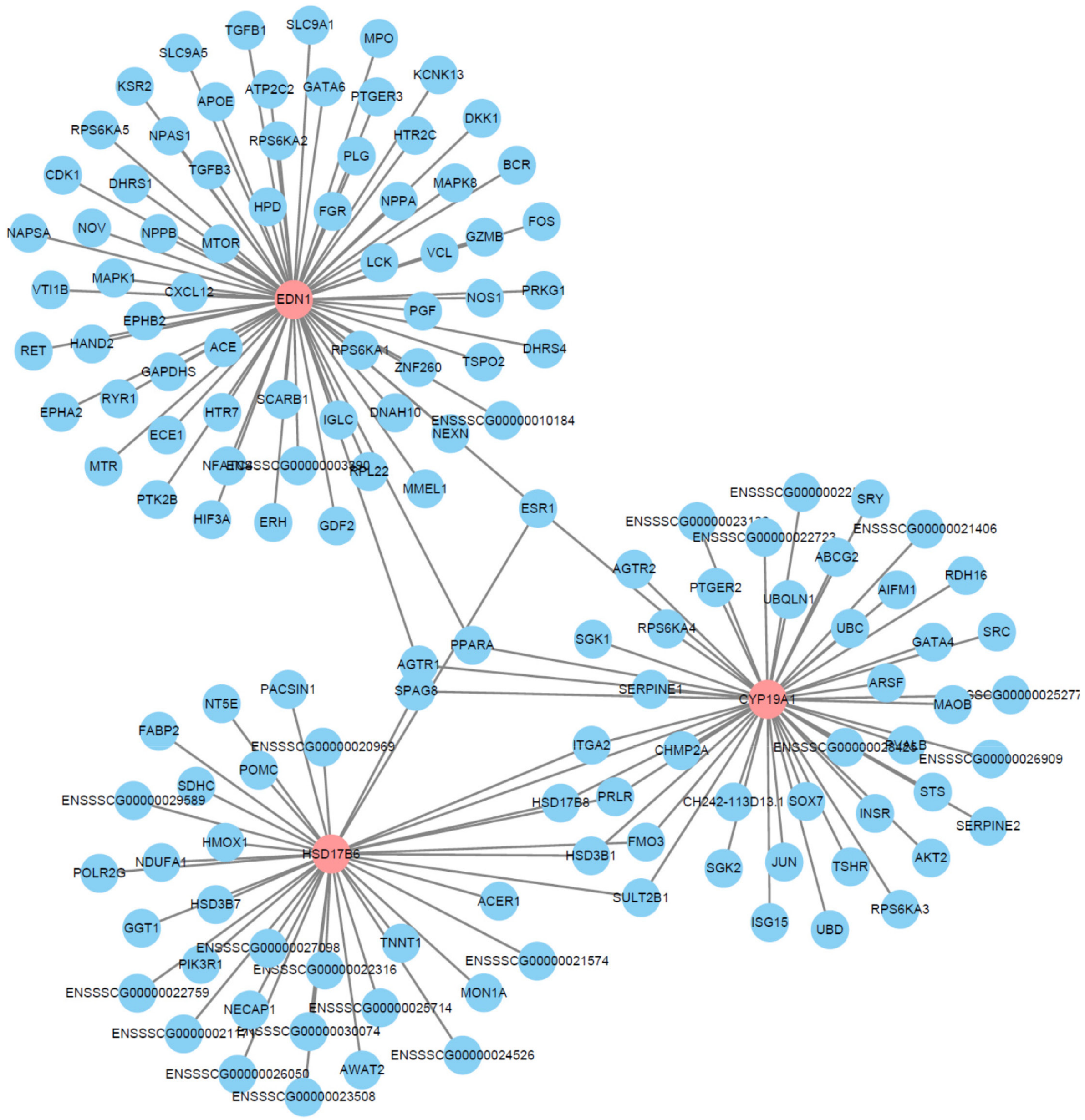


**Supplementary Figure 5: PPI network based on the STRING database to annotate functional interactions between steroidogenesis-related genes in ZEA-treatment groups.** We obtained 3 core differentially expression genes between control and ZEA-treated groups. Node degree ≥20 was selected as the threshold.

**Supplementary Table 1: Primers used for quantitative-PCR**

| Genes | Sequences of primers | Production(bp) | Genbank |
|---|---|---|---|
| *CYP19A1* | F:5′- GATGCTGCATTGTCACTGCT -3′ | 238 | KF_314685 |
| | R:5′-GGGAAGGTGTCACTCAAGGA-3′ | | |
| *FSHR* | F:5′- CGCTGGAAAGATGGCATACC-3′ | 155 | GU_990798 |
| | R:5′- GCTCACCTTCATGTAGCTGC -3′ | | |
| *HSD17β* | F:5′- TGATGGACTTCACCTGTGCT -3′ | 207 | NM_001185137 |
| | R:5′- GGCATGATCCCAGCATTCAG -3′ | | |
| *TGF-β* | F:5′-AGGAAACCCATAATGCCCCA-3′ | 208 | NM_214015.2 |
| | R:5′- TGGGCCCTATGGATAGCTG-3′ | | |
| *EDN-1* | F:5′- CTCCTTTGCGGTGATGATGG-3′ | 174 | NM_213882.1 |
| | R:5′- GGTGTGGACTTGGGTGTTTC-3′ | | |
| *IGF-1* | F:5′- TCCTGGAGTTGGTAGATTGCT-3′ | 224 | NM_214256.1 |
| | R:5′- ACGAATGTCTCTAGCAGCCA-3′ | | |
| *SFRP-2* | F:5′- GGCCTCAGGAATGGATAGCT-3′ | 165 | NM_001244395.1 |
| | R:5′- CCCCAAACATCACACCCAAG-3′ | | |
| *IER-3* | F:5′- AGATCGAAGGCCAAGGACAA-3′ | 154 | XM_001927551.3 |
| | R:5′- AGGGATGGAGAAAACTCGGG-3′ | | |
| *BDNF* | F:5′-CCTGGATGCTGCAAACATGT-3′ | 226 | NM_214259.2 |
| | R:5′-CCCTCCTTTGTGTACCCCAT-3′ | | |
| *Bax* | F:5′-ATGCGTCCAAGGAAGACTGAG-3′ | 198 | XM_003127290.4 |
| | R:5′-CCCCAGTTGAAGTTGCCATCAG-3′ | | |
| *Bcl-2* | F:5′-GCAGAGATGTCCAGTCAG-3′ | 166 | XM_003121700.4 |
| | R:5′-CACCGAACTCAAAGAAGG-3′ | | |
| *GAPDH* | F:5′-CCCCTTCATTGACCTCCACT-3′ | 157 | NM_001206359.1 |
| | R:5′-CCATTTGATGTTGGCGGGAT-3′ | | |
